# Preparation of Herbal Nano-Formulation-Assisted Mouth Paint Using Titanium Dioxide Nanoparticles and Its Biomedical Applications

**DOI:** 10.7759/cureus.48332

**Published:** 2023-11-05

**Authors:** Mohamed Rifaath, Shanmugam Rajeshkumar, Jayasree Anandan, Tharani Munuswamy, Sulochana Govindharaj

**Affiliations:** 1 Pharmacology/Nanobiomedicine, Saveetha Dental College and Hospitals, Saveetha Institute of Medical and Technical Sciences, Saveetha University, Chennai, IND; 2 Pharmacology, Saveetha Dental College and Hospitals, Saveetha Institute of Medical and Technical Sciences, Saveetha University, Chennai, IND

**Keywords:** biomedical applications, ginger, lemongrass, titanium dioxide nanoparticle, nanobiomedicine

## Abstract

Background

Mouth paint is a liquid oral care solution applied topically to the mouth, formulated to address oral health concerns like bacterial infections, inflammation, and bad breath. To improve the effectiveness of oral healthcare, nanomaterials are utilized in many dental products. Titanium dioxide nanoparticles (TiO_2_NPs) exert their antimicrobial effects through mechanisms like producing reactive oxygen species and direct interaction with microbial cells. The current study explored the antimicrobial, anti-inflammatory, and cytotoxic effects of the mouth paint prepared using TiO_2_NPs using an aqueous formulation of lemongrass and dry ginger.

Methods

Mouthpaint prepared using TiO_2_NPs synthesized using lemongrass and ginger was prepared and tested for potential applications. The antimicrobial activity of the prepared TiO_2_NPs-mediated mouth paint at different concentrations (25, 50, and 100 µL) against oral pathogens (*Streptococcus mutans, Enterococcus faecalis, Staphylococcus aureus,* and *Candida albicans)* was evaluated using the Agar well diffusion method. The anti-inflammatory activity of the produced mouth paint was examined using a bovine serum albumin denaturation assay and an egg albumin denaturation assay. The cytotoxic effect of the produced mouthpaint was analyzed using a brine shrimp lethality assay.

Results

Green synthesized TiO_2_NPs showed potent antimicrobial activity against the tested oral pathogens by exhibiting a zone of inhibition of 11 mm on Petriplate against *Staphylococcus aureus* and *Candida albicans* at 100 μL concentration. The prepared nanoparticles-mediated mouth paint possesses significant anti-inflammatory activity by effectively preventing the denaturation of bovine serum albumin, with a 74% inhibition at a concentration of 50 μL. The egg albumin denaturation assay showed a percentage inhibition of 80% at 50 μL. At the lowest concentration of 5 μL of the prepared mouth paint, 90% of the nauplii (egg-to-larvae stage of brine shrimp) remained alive after 48 hours.

Conclusion

The results showed that mouthpaint prepared using titanium dioxide nanoparticles synthesized using lemongrass and dry ginger formulations possessed significant antimicrobial activity and also displayed potential anti-inflammatory activity. The prepared mouth paint also displayed less toxicity, and hence, it can be used as an alternative to the commercially available synthetic mouth paint, which has more side effects.

## Introduction

Oral diseases encompass a spectrum of health conditions affecting structures within the mouth, including teeth, gums, the tongue, and related tissues [1]. The severity of these conditions can vary, and if left untreated, they may have significant implications for overall health. Factors such as poor oral hygiene, tobacco use, an unhealthy diet, and genetic predisposition can contribute to oral diseases [2]. Oral candidiasis, also known as thrush, is a fungal infection characterized by the excessive growth of *Candida* species, a yeast-like fungus, in the oral cavity [3]. Mouth paint is a liquid oral hygiene solution used for topical use within the oral cavity. Renowned for its aseptic and therapeutic qualities, mouth painting stands out as a convenient and efficient method [4].

Dental products incorporate nanomaterials to enhance the efficacy of oral healthcare. Nanotechnologies and nanomaterials have been widely used in the fields of medicine and nanomedicine for their various biomedical applications. Three primary application areas actively pursued are diagnosis, drug delivery, and regenerative medicine [5]. Nanoparticles find more applications in dentistry worldwide, including tooth whitening and coatings for dental materials, anti-sensitivity medications, and caries prevention [6]. In recent years, the integration of nanotechnology has paved the way for innovative and more effective mouth paint formulations [7]. Titanium dioxide nanoparticles (TiO2NPs) have been shown to possess antimicrobial properties, making them potentially useful in the fight against bacterial, viral, and fungal-caused oral diseases. This covers ailments like oral thrush, periodontal disease, and dental caries (cavities) [8].

Ayurveda highlights the medical properties of ginger, which is a significant herb known for its unique taste and scent [9]. Among the other bioactive compounds present in ginger, gingerol and shogoal are the two compounds thought to be responsible for their therapeutic effects from both biological and pharmacological perspectives. Scientific research has supported the use of ginger for dental health, as ginger extracts have well-known antibacterial, antifungal, anti-nausea, anticancer, and antiplaque properties. Ginger is also known for its indirect remineralization properties, contributing to its reputation for strengthening teeth. In summary, ginger offers various oral health benefits and is effective in the treatment of many dental conditions [10]. Lemongrass oil effectively eliminates microorganisms from the mouth and protects against tooth decay and gum diseases. Its astringent properties help strengthen the gums, thereby preventing gum disease and tooth decay. Additionally, the refreshing scent of lemongrass oil contributes to fresh breath. It can be incorporated into mouth rinses for plaque removal and prevention, effectively preventing cavity development [11]. Lemongrass can be used in mouth rinses and toothpaste to eliminate the biofilm that can eventually turn into plaque since it is good at cutting through the tough plaque biofilm [12]. It leads to increased antioxidant levels and reduced bacteria, aiding in the prevention and treatment of periodontitis [13].

The primary purpose of this study is to investigate and develop an innovative mouth paint formulation that holds the unique properties of TiO_2_NPs in combination with natural extracts from lemongrass and dry ginger. In this current research work, the prepared mouth paint was checked for antimicrobial activity against oral pathogens and also examined for its anti-inflammatory activity and cytotoxic effect.

## Materials and methods

Preparation of lemongrass and ginger formulation

Lemongrass leaves were collected from the terrace garden at Saveetha Dental College and Hospitals. The collected lemongrass was washed with double-distilled water to remove the impurities present in the leaves, shade-dried for 4-5 days, ground into a fine powder, and stored in an airtight container. Dry ginger powder was commercially bought from the local store located in Poonamallee. One gram of lemongrass leaf powder was measured and added to 50 mL of distilled water, and one gram of dry ginger powder was added to another 50 mL of distilled water. Both mixtures were heated using a heating mantle at 55°C for 15-20 minutes. Subsequently, these extracts were filtrated using Whatman No. 1 filter paper to remove the impurities. The resulting extracts were mixed and boiled using a heating mantle for 5-10 minutes. The resulting formulation was used for further processing.

Green synthesis of titanium dioxide nanoparticles

A quantity of 0.35 g of titanium oxide was measured in 50 mL of distilled water to make a titanium oxide solution. 50 mL of herbal formulation was added to the prepared titanium oxide solution. The reaction solution was kept in an orbital shaker at 160-180 RPM (rotation per minute) for 48 hours. After that, centrifugation was carried out at 8000 RPM (rotation per minute) for 10 minutes to segregate the pellet. Then the remaining supernatant was discarded, and the pellet was collected and kept in a hot air oven to make it into a powder. From there, 100 mg of nanoparticles were dissolved in 10 mL of distilled water, and the resulting solution was kept in the refrigerator at 4 oC for further use. A UV-Vis spectrophotometer (ESICO) was used to record the wavelength-dependent UV-Visible spectra of titanium dioxide nanoparticles within the 250-650 nm range. UV-visible spectroscopy and visual observation were used to confirm the synthesis of titanium dioxide nanoparticles.

Preparation of mouth paint

500 µL of TiO2NPs were added to 6 mL of propylene glycol. To that, 3.5 mL of glycerin was added. This mixture was stirred vigorously using a vortex mixer for 1-2 hours. The prepared mouth paint was tested for antimicrobial, anti-inflammatory, and cytotoxicity effects.

Antimicrobial activity

The antimicrobial activity of the prepared mouth paint was examined using the agar-well diffusion method against *Streptococcus mutans*, *Enterococcus faecalis*, *Staphylococcus aureus*, and *Candida albicans*. The agar-well diffusion method was employed to assess the antimicrobial effectiveness of the mouth paint. Mueller Hinton Agar was used for bacterial cultures, and Rose Bengal Agar for fungal cultures. Sterile plates were loaded with agar medium and left to solidify. Following that, wells measuring 9 mm in diameter were established using a sterilized polystyrene tip, and the test organisms were applied to the plates using swabs. Various concentrations of mouth paint (25, 50, and 100 µL) were introduced into separate wells. In the fourth well, commercial mouth paint (Clotrimazole) was added. Subsequently, the plates were placed in an incubator at 37°C for 24 hours. After the incubation period, the areas of inhibition were assessed and measured.

Anti-inflammatory activity

Bovine Serum Albumin Denaturation Assay (BSA Assay)

A BSA assay was used to evaluate the green-synthesized TiO2NPs-mediated mouth paint for its anti-inflammatory properties. 0.45 mL of bovine serum albumin was combined with 0.05 mL of various concentrations of mouth paint (10-50 µL). A period of 10 minutes of incubation at room temperature was maintained. The samples were kept at 55°C in a water bath for 30 minutes. The control group was dimethyl sulfoxide, whereas the standard group was diclofenac sodium. Spectrophotometric measurements at 660 nm were analyzed on the samples.

The percentage of protein denaturation was determined utilizing the following equation:

% inhibition= Absorbance of control - Absorbance of sample/Absorbance of control X 100

Egg Albumin Denaturation Assay

2.8 mL of phosphate buffer and 0.2 mL of fresh egg albumin were used to perform the egg albumin denaturation assay. The reaction mixture was supplemented with various concentrations (10-50 µL) of the green-synthesized TiO2NPs-mediated mouth paint. A pH adjustment of 6.3 was made. A period of 10 minutes of incubation at room temperature was maintained. The samples were kept at 55°C in a water bath for 30 minutes. Dimethyl sulfoxide served as the control, whereas diclofenac sodium served as the standard. The samples were examined using a spectrophotometer at 660 nm wavelength.

The percentage of protein denaturation was determined using the following equation:

% inhibition= Absorbance of control - Absorbance of sample/Absorbance of control X 100

Brine shrimp lethality assay

A saline solution was prepared by dissolving 2 g of iodine-free salt in 200 mL of distilled water. Following this, a six-well ELISA was utilized, and each well was filled with 10-12 mL of saline solution. In each well, 10 nauplii were gently introduced, and varying concentrations of TiO2NPs-mediated mouth paint (5, 10, 20, 40, and 80 µL) were added to each well. The commercial mouth paint (Clotrimazole) was added to each well in the same concentration. The plates were then placed for an incubation period of 24 hours.

After the incubation period, the ELISA plates were noted, and observations were recorded about the number of viable nauplii present in each well. The quantification of these counts was evaluated using the formula below:

Number of dead nauplii/number of dead nauplii+number of live nauplii×100

## Results

Visual observation and UV-visible spectroscopy

**Figure 1:**
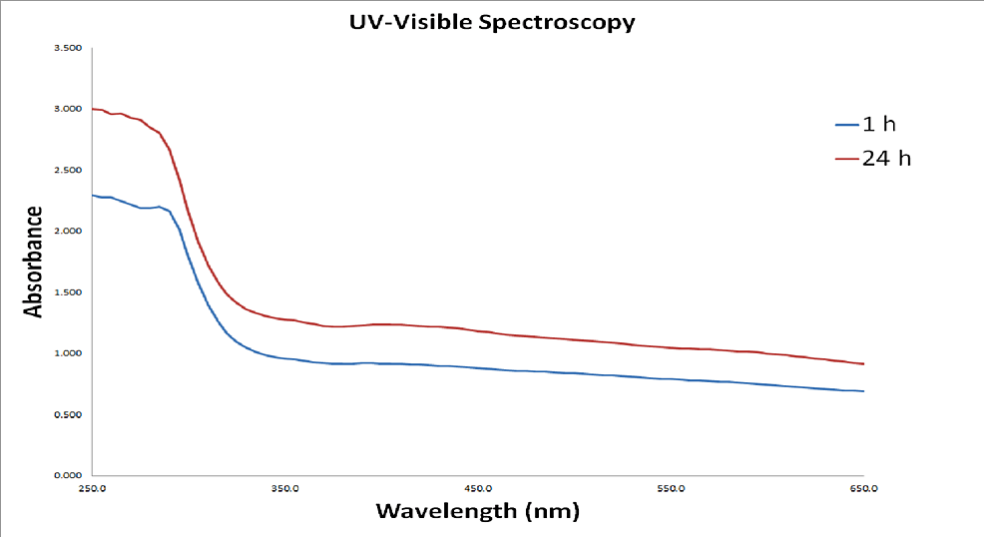
UV-visible spectra of C. citratus and Z. officinalis-mediated TiO2NPs The UV-visible spectrum of the biosynthesized TiO2NPs exhibits a peak at the wavelength of 285 nm, which further preliminary confirms the presence of the nanoparticles.

Visual observation is the preliminary tool for analyzing nanoparticle synthesis. After adding lemongrass and ginger formulations to the precursor titanium oxide solution, the initial color was observed to be pale yellow. The color change from pale yellow to pale greenish yellow was observed at the final stage. This preliminarily confirmation confirms the reducing and capping abilities of the lemongrass and ginger formulation. Figure 1 depicts the UV-visible spectral analysis for *C. citratus* and *Z. officinalis*-mediated TiO2NPs, revealing a characteristic surface plasmon resonance (SPR) peak with maximum absorbance at 285 nm, respectively. This finding provides preliminary confirmation of TiO2NPs formation. Both UV-visible spectroscopy and visual observation were used to preliminarily confirm the synthesis of TiO2NPs.

Antimicrobial activity

**Figure 2:**
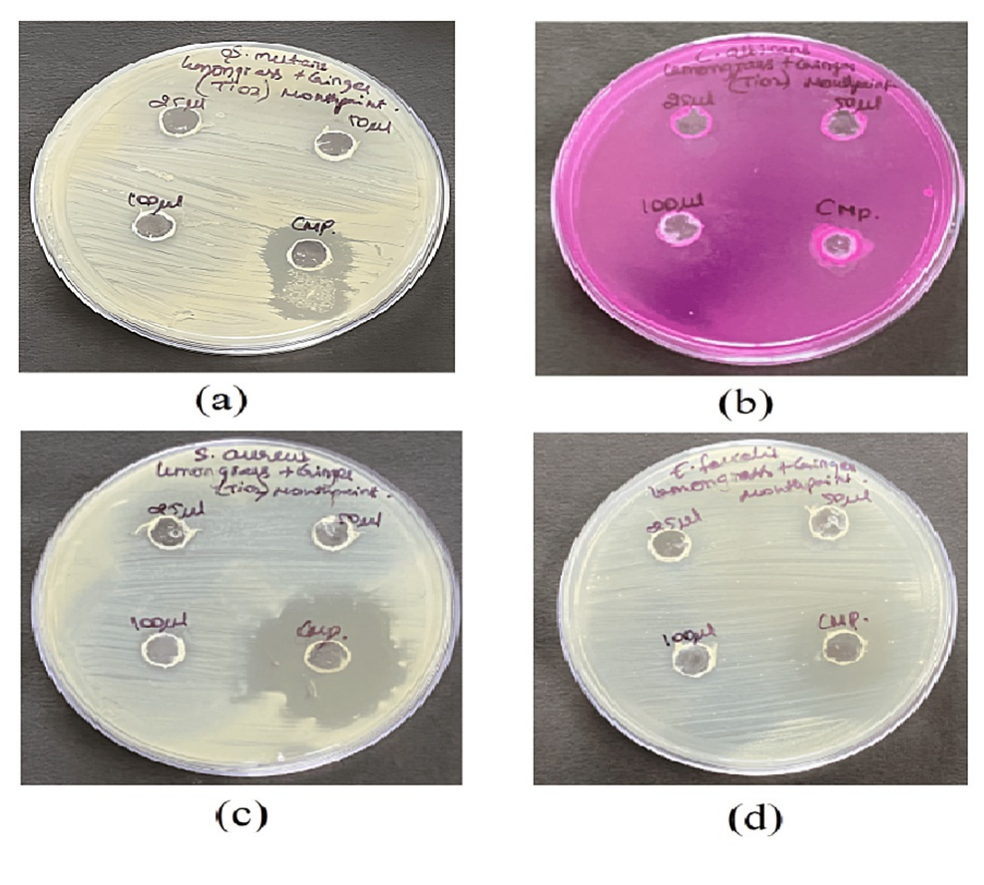
Antimicrobial activity of TiO2 nanoparticles-mediated mouth paint Images showing the antimicrobial activity of TiO2 nanoparticles-mediated mouth paint against four different oral pathogens (a) *S. mutans* (b) *C. albicans* (c) *S. aureus* (d) *E. faecalis*

The mouth paint prepared using lemongrass and ginger-mediated titanium dioxide nanoparticles was evaluated for its antimicrobial activity against oral pathogens. As shown in Figure 2, *Candida albicans* and *Staphylococcus aureus* organisms exhibited a higher zone of inhibition of 11 mm at a 100 μL concentration of prepared TiO2NPs-mediated mouth paint, followed by *Streptococcus mutans* and *Enterococcus faecalis*, and the antimicrobial activity of the prepared mouth paint is dose-dependent. But when compared to the commercial mouth paint, the green synthesized nanoparticles-mediated mouth paint showed less antimicrobial activity.

Anti-inflammatory activity

**Figure 3:**
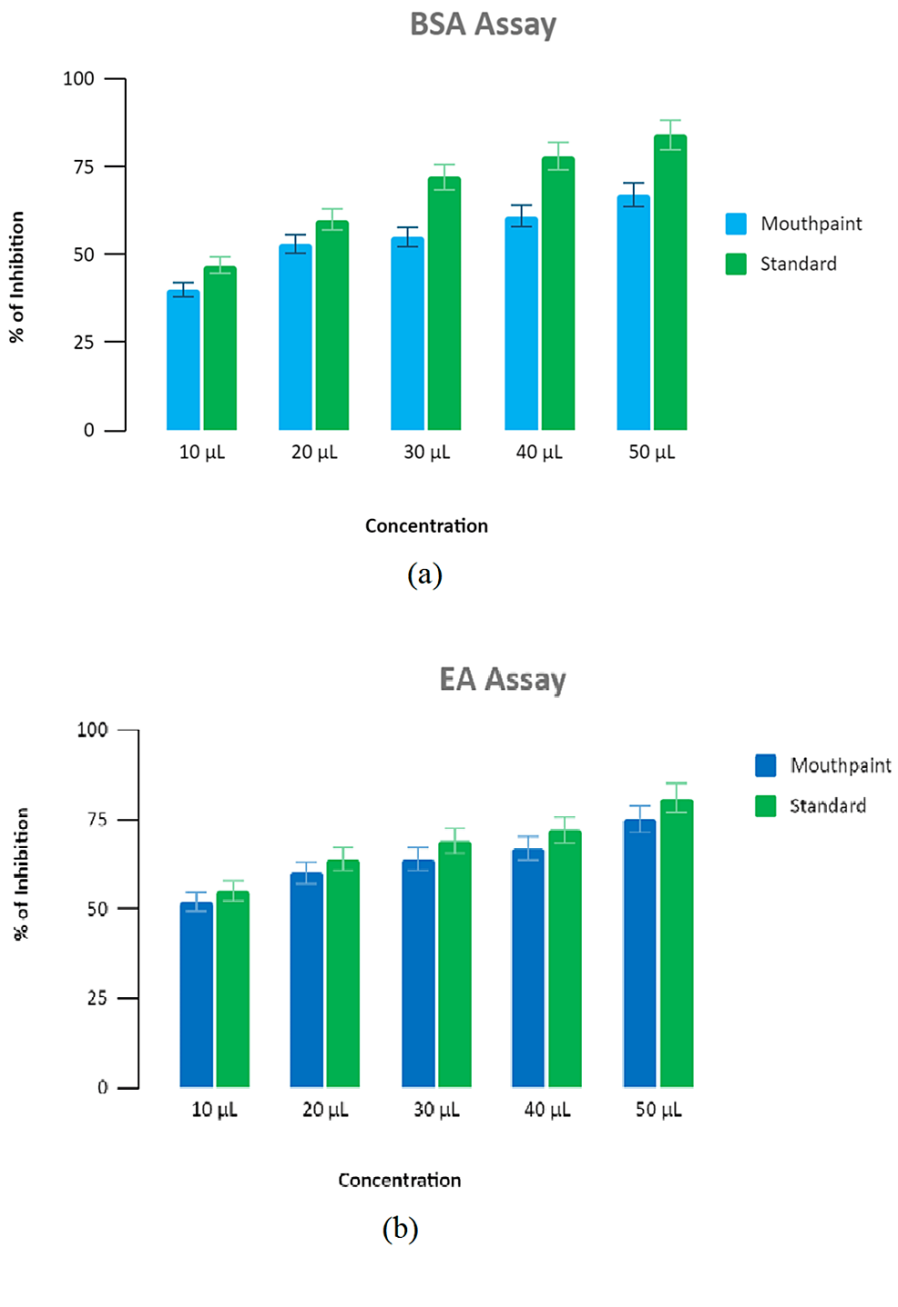
Anti-inflammatory activity (a) Bovine serum albumin denaturation assay (b) Egg albumin denaturation assay. The graph showing the anti-inflammatory activity of TiO2 nanoparticle-mediated mouth paint with BSA and EA assay

The anti-inflammatory activity of TiO2NPs-mediated mouth paint was assessed using two types of assays: the bovine serum albumin denaturation assay (Figure 3 (a)) and the egg albumin denaturation assay (Figure 3 (b)). In both of these assays, the minimum protein denaturation was observed at a high concentration of 50 µL by showing 74% (BSA assay) and 80% (EA assay) inhibition, respectively. However, the mouth paint showed slightly low protein denaturation when compared with standard diclofenac sodium. The anti-inflammatory activity, as determined by these two assays, demonstrated a dose-dependent effect. At all concentrations, the percentage of protein denaturation inhibition remained near the standard diclofenac sodium.

Cytotoxic effect

**Figure 4:**
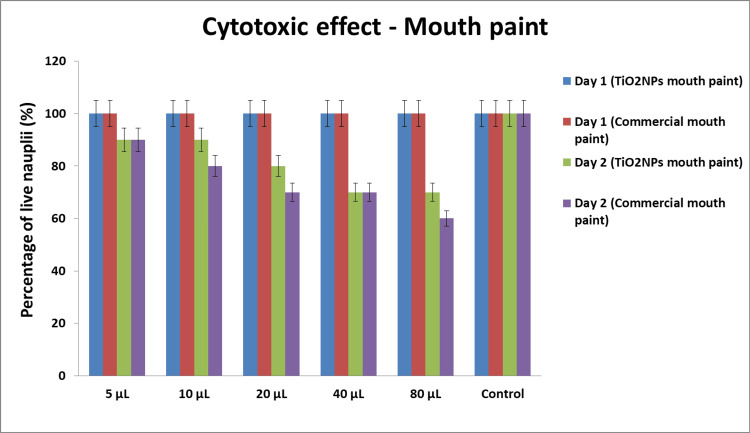
Cytotoxic effect of TiO2 nanoparticle-mediated mouth paint The graph represents the cytotoxic effect of TiO2 nanoparticle-mediated mouth paint estimated by the brine shrimp lethality assay (BSLA)

The cytotoxic effect of TiO2 nanoparticle-mediated mouth paint was determined by using a brine shrimp lethality assay with Artemia salina as a model. Different concentrations (5-80 µL) of the mouth paint were tested against the brine shrimps. The green synthesized nanoparticle-mediated mouth paint was cross-verified and compared with commercial mouth paint (Clotrimazole) and the control group. At a concentration of 5 µL, 90% of live nauplii were observed, while at 10 µL, 85% of live nauplii were observed. Subsequently, the 20 µL concentration showed 80% live nauplii, and the 40 µL showed 75% live nauplii. At the highest concentration of 80 µL, 60% live nauplii were observed on day 2. The control group showed 100% live nauplii on days 1 and 2. The green synthesized titanium dioxide nanoparticle-mediated mouth paint exhibited a less cytotoxic effect when compared to the commercial mouth paint, as shown in Figure 4. Animal ethics is not applicable to studying cytotoxic effects in the nauplii stage of brine shrimps.

## Discussion

The present study shows that the mouth paint prepared using TiO2NPs mediated through lemongrass and dry ginger has potent antimicrobial activity by displaying a zone of inhibition of 11 mm against *Staphylococcus aureus* and *Candida albicans* at a 100 μL concentration. However, the nanoparticle-mediated mouth paint showed slightly low antimicrobial activity compared to the commercial mouth paint. The prepared TiO2NPs-mediated mouth paint demonstrates significant anti-inflammatory activity, exhibiting a 74% inhibition in the bovine serum albumin denaturation assay at a concentration of 50 μL. Similarly, the egg albumin denaturation reveals an 80% inhibition at the same concentration. The prepared nanoparticles showed slightly less anti-inflammatory activity compared to the standard diclofenac sodium. At a concentration of 5 µL, 90% of live nauplii were present, and at a concentration of 80 µL, 60% of live nauplii were present. The commercial mouth paint is slightly more toxic compared to the green synthesized TiO2NPs-mediated mouth paint, which displays the compatibility of the mouth paint.

Scientists and researchers are continually searching for alternative therapeutic agents to traditional antibiotics to combat the challenge posed by multi-drug-resistant pathogens [14]. By regulating the morphology and crystal structure of TiO2NPs, the antibacterial effectiveness of the nanoparticles can be controlled. The best structure can be achieved through carefully examined surface features provided by shaped nanoparticles that improve antibacterial effectiveness, with results differing for particular bacterial strains [15]. The strong oxidizing power of TiO2NPs, which generates free radicals like hydroxyl and superoxide anion radicals, promotes the inactivation of microorganisms. This results in a reduction in the growth of various microorganisms, including both gram-positive and gram-negative bacteria [16].

In a previous study, TiO_2_NPs synthesized using *Calotropis gigantae* showed potential antifungal activity against *Aspergillus niger* [17]. In another study, the TiO_2_NPs prepared using *Luffa acutangula *leaf extract showed excellent antimicrobial activity against both the tested pathogenic bacteria and fungi [18]. Prior research has highlighted the strong antibacterial properties of TiO_2_NPs, and our findings align with these established outcomes, which show that TiO_2_NPs synthesized through lemongrass and ginger extract have the potential to serve as an excellent antimicrobial agent. In one study, caffeic acid-based titanium nanoparticles showed potential anti-inflammatory activity and excellent cytotoxic effect against A375 cancer cell lines [19], and in another study, TiO_2_NPs prepared using grape seed extract showed potent anti-inflammatory activity. In a previous study, curcumin-coated titanium dioxide nanoparticles prepared using *Myristica fragrans* showed potent anti-inflammatory activity in the hemolytic assay [20], and in another study, the green synthesized TiO_2_NPs using *Mucana pruriens L.* showed good antioxidant activity against DPPH free radicals and displayed significant anti-inflammatory activity in the albumin denaturation assay [21].

The results of previous research showed that the green synthesized TiO_2_NPs display significant anti-inflammatory activity, which correlates with this current research. The titanium dioxide nanoparticles prepared using lemongrass and ginger have biocompatibility, non-toxicity, and sustainability in addition to being safe and economical [22]. Overall, the cytotoxic activity outcomes demonstrated a relatively low toxicity rate of the mouth paint produced by TiO_2_NPs, which is consistent with the results of the present investigation. This shows that the mouth paint had a minimally cytotoxic effect on brine shrimp nauplii, indicating that it would be safe to use in the future.

Limitations

In the present studies, we completed several types of in-vitro analyses to evaluate the mouth paint mediated by TiO_2_NPs. Additional in vivo research, such as animal and clinical trials, will be helpful in better understanding its effects.

## Conclusions

Nanoparticle-based mouth paint preparation is a novel approach in dentistry. This study shows that lemongrass and ginger incorporated with TiO2NPs display potent antimicrobial and anti-inflammatory activity. However, the prepared mouth paint showed slightly low antimicrobial and anti-inflammatory activity compared to the standard and control. Yet, it shows a less cytotoxic effect compared to commercial mouth paint, which displays the biocompatibility of the green synthesized mouth paint. So, the green synthesized titanium dioxide nanoparticle-mediated mouth paint can be used to reduce the side effects caused by commercial synthetic mouth paint. Further research will be conducted, including in vivo studies and FDA clearance, as it is necessary before claiming the use of our green-synthesized mouth paint to reduce the side effects of commercial mouth paint. 
